# Task-related functional connectivity in autism spectrum conditions: an EEG study using wavelet transform coherence

**DOI:** 10.1186/2040-2392-4-1

**Published:** 2013-01-12

**Authors:** Ana Catarino, Alexandre Andrade, Owen Churches, Adam P Wagner, Simon Baron-Cohen, Howard Ring

**Affiliations:** 1 Cambridge Intellectual and Developmental Disabilities Research Group, Department of Psychiatry, University of Cambridge, Douglas House, 18b Trumpington Road, Cambridge, CB2 8AH, UK; 2 Institute of Biophysics and Biomedical Engineering, Faculty of Sciences, University of Lisbon, Campo Grande, 1749-016, Lisbon, Portugal; 3 University of South Australia, GPO Box 2471, Adelaide, SA 5001, Australia; 4 National Institute for Health Research (NIHR) Collaborations for Leadership in Applied Health Research and Care (CLAHRC) for Cambridgeshire and Peterborough, Peterborough, UK; 5 Autism Research Centre, Department of Psychiatry, University of Cambridge, Douglas House, 18b Trumpington Road, Cambridge, CB2 8AH, UK; 6 Cambridgeshire and Peterborough NHS Foundation Trust, Elizabeth House, Fulbourn Hospital, Fulbourn, Cambridge, CB21 5EF, UK

**Keywords:** Autism spectrum conditions, Interhemispheric coherence, Atypical connectivity, Wavelet transform coherence

## Abstract

**Background:**

Autism Spectrum Conditions (ASC) are a set of pervasive neurodevelopmental conditions characterized by a wide range of lifelong signs and symptoms. Recent explanatory models of autism propose abnormal neural connectivity and are supported by studies showing decreased interhemispheric coherence in individuals with ASC. The first aim of this study was to test the hypothesis of reduced interhemispheric coherence in ASC, and secondly to investigate specific effects of task performance on interhemispheric coherence in ASC.

**Methods:**

We analyzed electroencephalography (EEG) data from 15 participants with ASC and 15 typical controls, using Wavelet Transform Coherence (WTC) to calculate interhemispheric coherence during face and chair matching tasks, for EEG frequencies from 5 to 40 Hz and during the first 400 ms post-stimulus onset.

**Results:**

Results demonstrate a reduction of interhemispheric coherence in the ASC group, relative to the control group, in both tasks and for all electrode pairs studied. For both tasks, group differences were generally observed after around 150 ms and at frequencies lower than 13 Hz. Regarding within-group task comparisons, while the control group presented differences in interhemispheric coherence between faces and chairs tasks at various electrode pairs (FT7-FT8, TP7-TP8, P7-P8), such differences were only seen for one electrode pair in the ASC group (T7-T8). No significant differences in EEG power spectra were observed between groups.

**Conclusions:**

Interhemispheric coherence is reduced in people with ASC, in a time and frequency specific manner, during visual perception and categorization of both social and inanimate stimuli and this reduction in coherence is widely dispersed across the brain.

Results of within-group task comparisons may reflect an impairment in task differentiation in people with ASC relative to typically developing individuals.

Overall, the results of this research support the value of WTC in examining the time-frequency microstructure of task-related interhemispheric EEG coherence in people with ASC.

## Background

Autism Spectrum Conditions (ASC) are a set of pervasive neurodevelopmental conditions with an onset in early childhood and a wide range of lifelong symptoms. The core features of ASC include impairments in reciprocal social interactions, repetitive behaviors, a restricted range of interests and a variety of language disturbances, ranging from a complete absence of receptive and expressive speech to subtle problems of semantics and pragmatics [1]. In addition to these characteristic social and cognitive features, atypical patterns of perception and sensory integration are increasingly recognized as features of ASC [2-4].

Several explanatory models at the neurological level have been proposed to account for this range of features. The weak central coherence model [5] and the executive dysfunction model [6] are based on observations suggesting an etiological role for decreased neural connectivity [7], particularly with frontal regions [8]. Models of abnormal connectivity, including increased short-range connectivity and decreased long-range connectivity, have been proposed by several authors as a possible neurological substrate for the full range of behavioral and cognitive characteristics of ASC [9-13], [for recent reviews see [14,15].

Evidence to support the connectivity model comes from multiple methods of neuroanatomical and functional measurement. Several studies have shown abnormal trajectories of brain growth in ASC, specifically faster growth in the first two years followed by an asymptote between two and four years of age [16,17], [for reviews see [18,19]. It is thought that this difference in growth rates during the first years of life disrupts development of neural circuitry essential for higher order social, language and cognitive functions [18]. Of particular interest to the current study is the discovery of corpus callosum thinning in people with ASC [20,21]. According to previous investigations [22-24] white matter density appears to be decreased in the genu, rostrum and splenium of the corpus callosum in people with ASC, reflecting decreased interhemispheric structural connectivity in this group, relative to typically developing individuals. In addition, diffusion tensor imaging has shown decreased white matter integrity and connectivity in ASC [25-29], and cytoarchitectural analysis of brain samples has shown that ASC is associated with narrower mini-columns and decreased inter-column spacing in frontal and temporal areas [30], which are likely to disrupt the formation of long-range connections between neural networks, impairing systems involved in top-down control and integration of information [30].

Moving from structural to functional analysis, dynamic causal modeling [31] has revealed decreased connectivity in ASC during tasks involving the interpretation of the affective meaning of abstract shapes [32], actions [33] and perceiving emotionally expressive faces [30,34]. Finally, the analysis of electroencephalogram (EEG) signals has provided evidence of long-range under-connectivity along with short-range over-connectivity in ASC as reflected by EEG signal coherence between different electrode sites [35]. In addition, a previous study from our research group on EEG complexity in ASC [36] showed evidence of decreased signal complexity in parietal and occipital sites in individuals with ASC, compared to typically developing individuals, suggesting decreased neural connectivity, possibly associated with relatively reduced long-range temporal correlations in EEG in these regions.

Coherence is a measure of the level of synchronization of activity between different neural populations, with high coherence reflecting greater synchronization and, hence, greater functional integration due to either direct cortico-cortical connections or indirect cortical-subcortical-cortical connections [37]. Coherence between different EEG electrodes has traditionally been calculated using a Fast Fourier Transform (FFT) to determine the power in discrete frequencies across a large period of time. Using this approach, decreased intrahemispheric coherence in ASC has been found between frontal and other scalp sites, along with increased coherence within frontal and temporal [38], lateral frontal [35] and occipital sites [39]. Decreased interhemispheric coherence in ASC has been found across frontal and parietal sites [40] and across occipital sites [41].

While this method has delivered novel insights into functional connectivity between different brain regions in ASC, there is a limitation to the use of FFT in coherence analysis. As coherence is calculated over a relatively long time period (usually one second), this methodology is incapable of providing information about the temporal structure of coherence [42]. High temporal resolution is an advantage that EEG has over other imaging methodologies and knowledge of the time points at which coherence is heightened or reduced is essential for an understanding of complex brain dynamics [42]. A method of analyzing coherence at different time points will allow for the analysis of coherence related to the perception of specific stimuli, rather than during a period of rest [35,38,40] or sleep [39].

This can be achieved using Wavelet Transform Coherence (WTC) [42]. This is a technique for the analysis of coherence between electrodes as a function of time. WTC performs a time-frequency analysis of the signals by transforming the original signal using a wavelet function with a characteristic time *t* and frequency *f*. The Morlet wavelet is one of the most popular choices of wavelet. The wavelet coherence between two signals can then be calculated for any time-frequency bin. Hence, WTC retains the high temporal resolution available from EEG data and has the advantage of generating coherence values for the entire time-frequency spectrum, allowing for the analysis of coherence related to particular events in time, such as the presentation of visual stimuli.

The aims of the current study are as follows: First, to test the hypothesis that participants with ASC will manifest reduced interhemispheric coherence, calculated using WTC, when compared to a group of typically developing controls. This hypothesis arises out of earlier reports of decreased interhemispheric coherence measured using FFT. Second, building on the temporal and frequency resolution of the WTC approach, to test whether there are specific effects on group differences in coherence related to task and brain location, for specific frequency bands or time periods. Specifically, we will investigate these effects during the performance of a visual matching task involving social (images of faces) and non-social (images of chairs) stimuli. Based on various reports of facial processing impairments in people with ASC [8,34,43,44], and given the results from an event-related potential (ERP) investigation using the same paradigm as the current study [45], we hypothesize that while the control group will present different coherence profiles for the face and the chair task, such task differentiation will be absent in the ASC group, as shown by a lack of differences in coherence between tasks in this group.

## Methods

### Ethics

This study was approved by the Psychology Research Ethics Committee at the University of Cambridge and all participants gave informed written consent.

### Participants

Fifteen patients with ASC and 15 typical controls were recruited for this study. All ASC participants were diagnosed by a clinical psychologist or psychiatrist experienced with the diagnosis of ASC based on international criteria [1]. Exclusion criteria for ASC participants were uncorrected impairment in eyesight, impaired hand movement, or a personal or family history of any psychiatric or genetic condition apart from ASC. Exclusion criteria for control participants were similar, with the addition of any personal or family history of an ASC. All participants were male and right-handed, as measured by the Edinburgh Handedness Inventory [46].

Participants were administered the Wechsler Abbreviated Scale of Intelligence (WASI; [47]) for Intelligence Quotient (IQ) assessment and the Autism Spectrum Quotient (AQ; [48]). Higher scores on the AQ reflect a greater number of autistic traits. The ASC group (mean = 35, SD = 7) scored significantly higher than the control group (mean = 16, SD = 7, F_1, 28_ = 57.351; *P* <0.0005) in line with earlier studies. The participant groups were matched for age and IQ. The demographic details of participants along with their IQ and AQ scores are presented in Table 1.

**Table 1:** Participants’ characteristics

**Participants**’ **characteristics**							
	**Controls** (**n** = **15**)			**ASC** (**n** = **15**)			**Group comparison**
	**Mean**	**SD**	**Range**	**Mean**	**SD**	**Range**	
Age	29	4	21 to 37	31	6	23 to 42	F_1, 29_ = 0.961 ; *P* = 0.335
Verbal IQ ^a^	114	16	77 to 133	119	11	101 to 134	F_1, 28_ = 1.068 ; *P* = 0.310
Performance IQ ^a^	119	11	93 to 134	115	14	93 to 132	F_1, 28_ = 0.696 ; *P* = 0.412
Full-Scale IQ ^a^	119	14	93 to 134	119	13	98 to 136	F_1, 28_ = 0.007 ; *P* = 0.936
AQ ^b^	16	7	4 to 27	35	7	21 to 46	F_1,28_ = 57.351 ; *P* <0.0005

Age, verbal IQ, performance IQ and full-scale IQ for each group; (a) IQ scores were not available for one control participant; (b) Autism Quotient (AQ) scores were not available for one control participant (different from - a). SD, standard deviation.

### EEG recording

EEG data were acquired as part of an ERP protocol [45] using 28 standard scalp electrodes placed in accordance with the International 10–20 System [49]. The reference electrode was the tip of the nose with ground at Fpz. Eye-movements were monitored using bi-polar channels with electrodes above and below the left eye (vertical electro-oculogram) and 1 cm from the outer canthus of each eye (horizontal electro-oculogram). Impedances at all sites were maintained below 5kΩ. EEG data were obtained at a sampling frequency of 1,000 Hz, with a 0.1 to 50 Hz input bandpass filter, and using a 32-channel Synamps apparatus (Compumedics Neuroscan, Charlotte, NC, USA). Segments containing ocular, muscular movement and other artefacts were manually selected and removed from the data.

The EEG was recorded while participants performed a face and chair detection task. They were seated in a darkened room approximately 60 cm from a computer screen, on which stimuli were presented. The stimuli consisted of 30 photographs of neutral faces (15 male, 15 female) and 30 photographs of chairs. All stimuli were edited in Photoshop CS3 (http://www.adobe.com), transformed to grayscale, mounted on a white background, equated for average luminance and contrast, and resized to 5 x 7 cm. Participants viewed two blocks of stimuli between which only the order of the images varied. In each block, all 60 pictures (30 faces, 30 chairs) were presented three times pseudo-randomly without immediate repetition. Each image was presented for 500 ms, with an interstimulus interval that varied randomly between 1,200 ms and 1,400 ms. Thus each block lasted for about 5.5 minutes. In one of the blocks, the subject’s attention was directed to the photographs of chairs, and in the other block their attention was directed to the faces. To do this, 10 images of faces (5 male, 5 female) and 10 images of chairs were inserted as immediate repetitions. At the start of each block, participants were asked to attend to one of the categories of stimulus (faces or chairs) and to press a response button whenever they saw an immediate repetition of an image of that category, while ignoring all stimuli in the other category. The purpose of this instruction was to direct the participants’ attention to a given category. Response times and accuracy were measured for each participant. Each block began with a practice run of 10 stimuli. The order of the two blocks, the attended category and the hand used to respond were counterbalanced across participants. Participants rested for approximately 5 minutes between blocks.

### Signal analysis

Epochs were extracted from artefact-free sections of the EEG recordings using the SCAN software package (Compumedics, Neuroscan), for two distinct tasks – for the face task, epochs chosen included those where a picture of a face was presented, when the subjects’ attention was directed to faces; for the chair task, selected epochs included those where the presentation of a picture of a chair was made, when the subjects’ attention was directed to chairs. Due to the presence of artefacts, some epochs (from the initial number of 90) had to be excluded from the analysis. Despite this, the number of epochs included in the analysis did not differ significantly between groups (mean ASC = 81, SD = 8, mean control = 81, SD = 8, F_1, 29_ = 0.016, *P* = 0.901) or tasks (chair task: mean ASC = 81, SD = 9, mean control = 80, SD = 9, F_1, 29_ = 0.183, *P* = 0.672; face task: mean ASC = 81, SD = 8, mean control = 83, SD = 7, F_1,29_ = 0.569, *P* = 0.457). The time interval for each epoch was from 0 to 400 ms post-stimulus presentation. The WTC algorithm was applied to each epoch separately, for a frequency interval between 5 and 40 Hz. Coherence maps for the entire time-frequency space (0 ms to 400 ms post-stimulus onset, 5 Hz to 40 Hz) were calculated by averaging coherence values across all epochs. Analyses were run separately for the chairs and for the faces tasks. Electrodes Fp1, Fp2 and Fz were excessively affected by eye movement artefacts and were removed from the analysis. Technical problems affected electrodes F3 and O2 during data acquisition for some participants. Therefore, electrode pairs F3/F4 and O1/O2 were also excluded from the analysis.

Coherence maps were calculated, and statistical group and task comparisons were undertaken for available interhemispheric electrode pairs; F7-F8, FT7-FT8, T7-T8, TP7-TP8 and P7-P8. These pairs were included based on previous reports of atypical interhemispheric neural connectivity in people with ASC, in the context of visual or face processing tasks [40,41,45].

### Wavelet transform coherence (WTC)

Let *x* and *y* be two stationary signals. Let S_xx_ and S_yy_ denote the autospectral densities (that is, the Fourier transform of the autocorrelation function) of x and y, respectively, and S_xy_ be the cross-spectral density between x and y. The coherence between waveforms x and y can then be defined, at the frequency of interest f, as [42]:

(1) $$\varrho \left(f\right)=\frac{\left|S_{\mathrm{xy}}\left(f\right)\right|}{{\left[S_{\mathrm{xx}}\left(f\right).S_{\mathrm{yy}}\left(f\right)\right]}^{1/2}}\text{.}$$

However, this theoretical value of coherence can only be computed for waveforms of infinite duration. In real situations, with finite time-series, the coherence value is computed through approximation – the finite time-series x and y are divided into N overlapping segments, x_j_ and y_j_, j = 1, …, N. Each segment is multiplied by a weighting function (for example, Hamming window), and for each weighted segment the Fourier spectra, x’_j_ (f) and y’_j_ (f) are computed. For each segment, the cross-spectrum coefficient is calculated using the Fourier spectra:

(2) $$c_{j}\left(f\right)=x{'}_{j}\left(f\right).y{'}_{j}^{*}\left(f\right)\text{.}$$

The cross-spectral density estimation is defined by the average of the coefficients c_j_ over the *N* segments:

(3) $$S_{\mathrm{xy}}\left(f\right)\approx {\tilde{S}}_{\mathrm{xy}}\left(f\right)=\frac{1}{N}\sum_{j=1}^{N}c_{j}\left(f\right)=\frac{1}{N}\sum_{j=1}^{N}x{'}_{j}\left(f\right).y{'}_{j}^{*}\left(f\right)\text{.}$$

The same approximation can be done for the autospectral densities:

(4) $${\tilde{S}}_{\mathrm{xx}}\left(f\right)=\frac{1}{N}\sum_{j=1}^{N}x{'}_{j}\left(f\right).x{'}_{j}^{*}\left(f\right)\text{,}$$

(5) $${\tilde{S}}_{\mathrm{yy}}\left(f\right)=\frac{1}{N}\sum_{j=1}^{N}y{'}_{j}\left(f\right).y{'}_{j}^{*}\left(f\right)\text{,}$$

leading to an estimation of coherence defined as

(6) $$\tilde{\varrho }\left(f\right)=\frac{\left\|{\tilde{S}}_{\mathrm{xy}}\left(f\right)\right\|}{{\left[{\tilde{S}}_{\mathrm{xx}}\left(f\right).{\tilde{S}}_{\mathrm{yy}}\left(f\right)\right]}^{1/2}}\text{.}$$

However, most physiological signals are non-stationary, and in this case methods based on simple Fourier analysis are inadequate; the weighted segments would correspond to multiple sub-processes with different spectral properties, and averaging the spectral estimates of these segments would be meaningless [42].

In an attempt to improve the temporal resolution of coherence calculations, and to be able to study the time-course of coherence, alternative algorithms were created. Wavelet Transform Coherence (WTC) analysis overcomes the problem of non-stationarity by providing a time-frequency analysis of the coherence between two time-series x and y [42,50]. The current study employed a freely distributed algorithm for WTC analyses [51].

In WTC analysis, each signal x and y is wavelet transformed, that is, correlated with a wavelet function, which is a complex valued function with zero average [50]. Although there are many wavelet functions (for example, Paul, Mexican hat, Meyer), one of the most common wavelets used for computing coherence in physiological signals, and also the one we used in this investigation, is the Morlet wavelet [42,52]. The Morlet wavelet consists of the product of a sinusoidal wave at frequency f and a Gaussian function centered at time τ and with standard deviation σ (inversely proportional to frequency f), and can be defined as [42]:

(7) $$\Psi_{\tau }{{}_{,}}_{f}\left(u\right)=\sqrt{f}e^{i2\mathrm{\pi f}\left(u-\tau \right)}e^{-\frac{{\left(u-\tau \right)}^{2}}{\sigma^{2}}}\text{.}$$

The number of cycles of the Morlet wave is the same for all frequencies. The Morlet wavelet used in the current study was defined to have 4 cycles. This provides a good trade-off between noise and temporal frequency of the coherence estimate, and is consistent with the methods employed by previous studies [42,52]. As the standard deviation of the Gaussian function is inversely proportional to frequency f, the wavelet will be narrower in time as frequency increases, that is, the temporal resolution of the coherence estimate improves when frequency increases.

The wavelet transform of a given signal x at time τ and frequency f is therefore given by:

(8) $$W_{x}\left(\tau ,f\right)=\int_{-\infty }^{+\infty }x\left(u\right).\Psi_{\tau ,f}^{*}\left(u\right)\mathrm{du}\text{.}$$

From this we can define the wavelet auto and cross-spectral density, respectively, as

(9) $$SW_{\mathrm{xx}}\left(t,f\right)=\int_{t-\delta /2}^{t+\delta /2}W_{x}\left(\tau ,f\right).W_{x}^{*}\left(\tau ,f\right)\mathrm{d\tau}$$

and

(10) $$SW_{\mathrm{xy}}\left(t,f\right)=\int_{t-\delta /2}^{t+\delta /2}W_{x}\left(\tau ,f\right).W_{y}^{*}\left(\tau ,f\right)\mathrm{d\tau}\text{,}$$

where δ is a scalar that defines the temporal resolution of coherence estimates. In wavelet coherence, δ is dependent on frequency so that *δ* ∝ 1/f. This means that δ is smaller for high frequencies, that is, the temporal resolution of the coherence estimate improves when frequency increases. In the current study, δ values were determined by the WTC algorithm used [51,53].

Analogous to ordinary Fourier-based coherence, wavelet coherence is defined at time t and frequency f by:

(11) $$\mathrm{WCo}\left(t,f\right)=\frac{\left|SW_{\mathrm{xy}}\left(t,f\right)\right|}{\sqrt{SW_{\mathrm{xx}}\left(t,f\right).SW_{\mathrm{yy}}\left(t,f\right)}}\text{,}$$

where WCo(t,f) takes values between 0 (no coherence) and 1 (maximum coherence), and the time resolution of the estimated coherence is inversely proportional to the frequency in which it is computed [42,52].

### Power analysis

In order to investigate possible group differences in power spectra of the signals analyzed, a wavelet power analysis was performed for each participant. Raw data, free of artefacts, were wavelet transformed using a four-cycle Morlet wavelet. The power of the wavelet transformed signal was then calculated for four standard band frequencies: theta (5 to 8 Hz), alpha (8 to 13 Hz), beta (13 to 30 Hz) and gamma (30 to 40 Hz).

### Statistical analysis

Statistical analyses were carried out using SPSS Statistics v17.0 (SPSS Inc, Chicago, USA) and the statistical analysis package R (version 2.13.0, R Foundation for Statistical Computing, Vienna, Austria). The alpha significance values were set at 0.05.

To test for differences in behavioral results, a 2-way repeated-measures analysis of variance (ANOVA) was done for accuracy and response time, with group (ASC vs. controls) as a between-subjects factor, and task (chairs vs. faces) as a within-subjects factor. In order to reduce the skewness in the distributions, response time data were transformed using a logarithmic function (f(x) = ln(x)) and proportional accuracy was transformed using an arcsin function (f(x) = arcsin(√x)) [54].

The statistical software package R was used to run Mann–Whitney comparison analyses over the full time-frequency range (36 frequency points - 5 to 40 Hz - by 401 time points - 0 to 400 ms post-stimulus onset), to assess significant differences in WTC values between groups (ASC vs. controls) and tasks (chairs vs. faces), for all electrode pairs (F7-F8, FT7-FT8, T7-T8, TP7-TP8 and P7-P8). This is consistent with previous studies applying the WTC algorithm to the analysis of EEG data [52]. Additionally, correction for multiple comparisons was performed using a False Discovery Rate (FDR) algorithm implemented within the R software package [55,56]. Significant group differences are presented in time-frequency maps, where *P*-values smaller than 0.05 are represented in a gray scale.

To test for group differences in EEG power spectra a 4-way repeated-measures ANOVA was used, with group as a between-subjects factor, and task, electrode and frequency band (4 frequency bands: theta, alpha, beta, gamma) as within-subjects factors. The Greenhouse-Geisser adjustment was applied to the degrees of freedom for all analyses.

## Results

### Behavioral performance

Both groups performed both tasks with high levels of accuracy (mean accuracy (out of 10) for the chair task: controls = 9.73, SD = 0.59, ASC = 9.60, SD = 0.91; mean accuracy (out of 10) for the face task: controls = 9.73, SD = 0.46, ASC = 8.80, SD = 1.47). Regarding accuracy, a significant effect of task was found (F_1, 28_ = 4.898, *P* = 0.035), with lower accuracy for the face than for the chair task. No other significant group effects or group by task interactions were found, though the group-by-task interaction approached significance (F_1, 28_ = 3.661, *P* = 0.066). There were no significant group or task effects or interactions with response time. The accuracy and response times of participants are reported in Table 2.

**Table 2:** Behavioral results

	**Behavioral results**				
	**Controls** (**n** = **15**)		**ASC** (**n** = **15**)		**Group comparison**
	**Mean**	** SD**	** Mean**	** SD**	
**Chair task:**					
Accuracy (out of 10)	9.73	0.59	9.60	0.91	F_1, 29_ = 0.071 ; *P* = 0.791
Response time (ms)	479.01	83.43	514.10	70.47	F_1, 29_ = 1.907 ; *P* = 0.178
**Face task:**					
Accuracy (out of 10)	9.73	0.46	8.80	1.47	F_1, 29_ = 4.566 ; *P* = 0.041
Response time (ms)	493.12	88.46	515.34	84.36	F_1, 29_ = 0.572 ; *P* = 0.456

Accuracy (out of 10) and response times (in ms) for both tasks, for each group. *SD* standard deviation.

### WTC analysis

**Figure 1:**
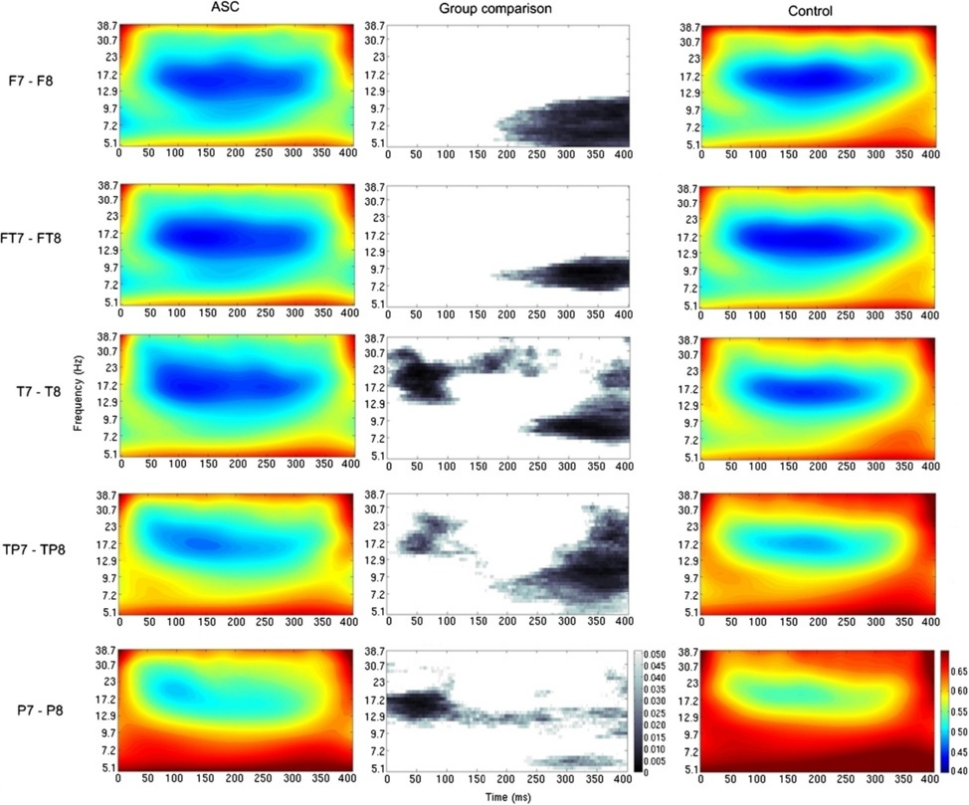
**Mann–Whitney group comparison of interhemispheric coherence for the chairs task (uncorrected *P*-values).** The colored graphs represent values of wavelet coherence for the ‘chairs’ task, for every time-frequency point and for each group (ASC: left column, controls: right column) and each pair of electrodes (different rows); areas in blue represent regions of low coherence in the time-frequency spectrum, while areas in red represent regions of high coherence. The graphs in the middle column represent the statistical group comparison for each electrode pair; areas shaded gray represent regions of the time-frequency spectrum where there is a significant decrease of coherence for the ASC group compared to the control group, at *P*_uncorrected_ <0.05. No areas were found where there was a significant increase in coherence for the ASC group compared to the control group.

**Figure 2:**
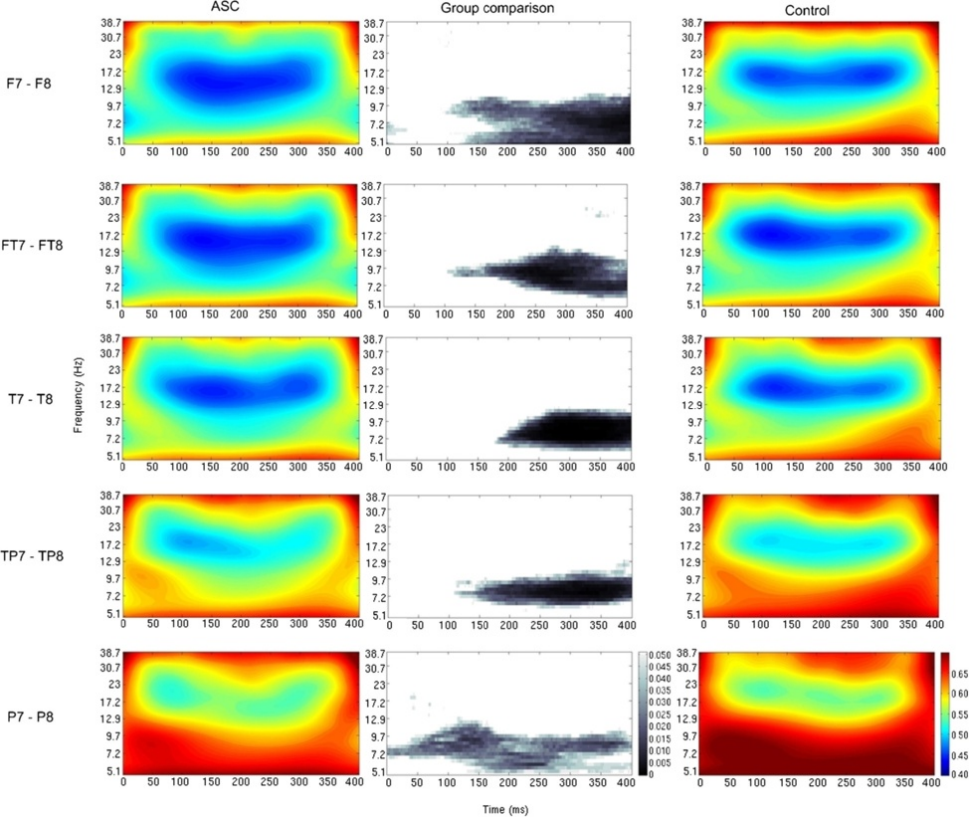
**Mann–Whitney group comparison of interhemispheric coherence for the faces task (uncorrected *P*-values).** The colored graphs represent values of wavelet coherence for the ‘faces’ task, for every time-frequency point and for each group (ASC: left column, controls: right column) and each pair of electrodes (different rows); areas in blue represent regions of low coherence in the time-frequency spectrum, while areas in red represent regions of high coherence. The graphs in the middle column represent the statistical group comparison for each electrode pair; areas shaded gray represent regions of the time-frequency spectrum where there is a significant decrease of coherence for the ASC group compared to the control group, at *P*_uncorrected_ <0.05. No areas were found where there was a significant increase in coherence for the ASC group compared to the control group.

Mann–Whitney analyses of interhemispheric coherence revealed various patterns of significant group differences. There were patterns of significantly decreased coherence for the ASC group when compared to the control group, for both tasks (chairs and faces) and for all the electrode pairs studied, at *P*_uncorrected_ <0.05 (Figures 1 and 2). Across both tasks this relatively decreased coherence in the ASC group was observed largely for frequencies below about 13 Hz and, except at the parietal electrode pair, only at times later than about 150 ms post stimulus onset. For this time-frequency region, no significant correlations were found between coherence and IQ or AQ scores, for either group. It is interesting to note, however, that for the ASC group the correlations between coherence and AQ scores were negative, for both tasks and all electrode pairs.

**Figure 3:**
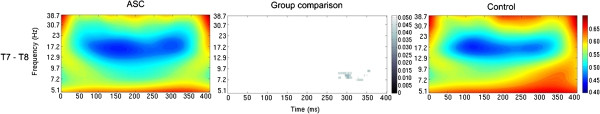
**Mann–Whitney group comparison of interhemispheric coherence, corrected for multiple comparisons using False Discovery Rate (FDR).** The colored graphs represent values of wavelet coherence for the ‘faces’ task, for every time-frequency point, for each group (ASC: left column, controls: right column) and for electrode pair T7-T8; areas in blue represent regions of low coherence in the time-frequency spectrum, while areas in red represent regions of high coherence. The graph in the middle represents the FDR-corrected statistical group comparison for electrode pair T7-T8; areas shaded gray represent regions of the time-frequency spectrum where there is a significant decrease of coherence for the ASC group compared to the control group, at *P*_FDR-corrected_ <0.05. No areas were found where there were significant group differences after FDR correction for any other electrode pairs or task.

After correcting for multiple comparisons, the only significant group differences in interhemispheric coherence were at electrode pair T7-T8 and only for the faces task. These group differences remained significant (*P*_FDR-corrected_ <0.05) for a time-frequency window around 300 ms post-stimulus onset and between 7 and 10 Hz (Figure 3). No regions of increased coherence for the ASC group were found compared to the control group, for any task or electrode pair, at *P*_uncorrected_ <0.05.

**Figure 4:**
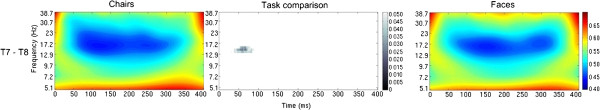
**Mann–Whitney task comparison of interhemispheric coherence for the ASC group (uncorrected *P*-values).** The colored graphs represent values of wavelet coherence for the ASC group, for every time-frequency point, for each task (Chairs: left column, Faces: right column) and for electrode pair T7-T8; areas in blue represent regions of low coherence in the time-frequency spectrum, while areas in red represent regions of high coherence. The graph in the middle represents the statistical task comparison for electrode pair T7-T8; areas shaded gray represent regions of the time-frequency spectrum where there is a significant decrease of coherence for the chairs task when compared to the faces task, at *P*_uncorrected_ <0.05. For the ASC group, no areas of significant differences in coherence between tasks were found for any other electrode pairs.

**Figure 5:**
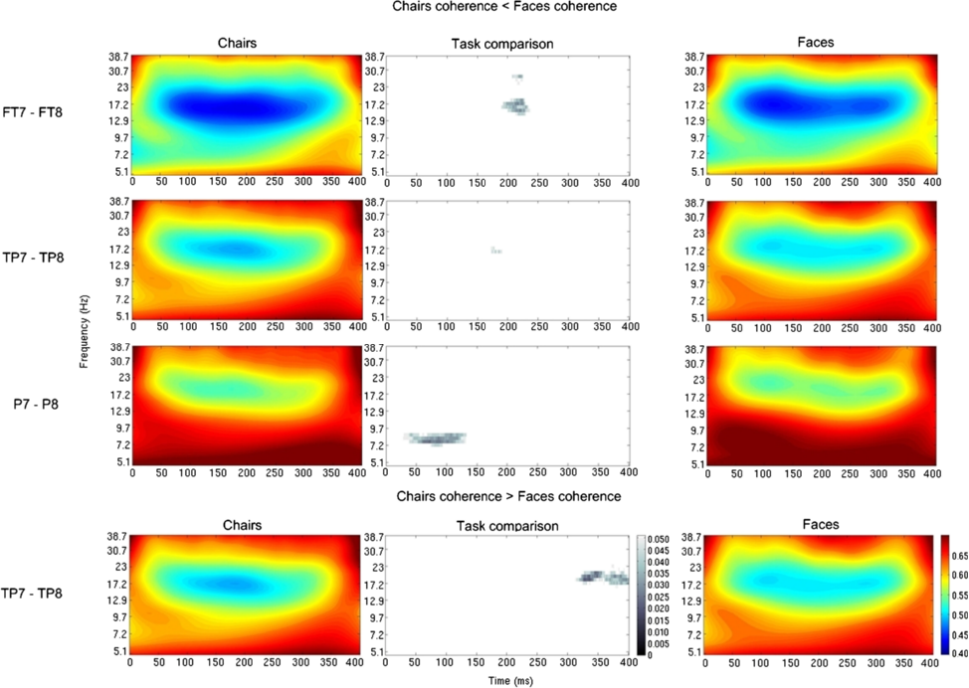
**Mann–Whitney task comparison of interhemispheric coherence for the control group (uncorrected *P*-values).** The colored graphs represent values of wavelet coherence for the control group, for every time-frequency point and for each task (Chairs: left column, Faces: right column), for different electrode pairs (each row) and directions (decreased coherence for chairs task relative to faces task on first three rows, increased coherence for chairs relative to faces task on last row); areas in blue represent regions of low coherence in the time-frequency spectrum, while areas in red represent regions of high coherence. The graphs in the middle column represents the statistical task comparison for different electrode pairs; areas shaded gray represent regions of the time-frequency spectrum where there is a significant difference in coherence between the chairs and the faces task, at *P* (uncorrected) <0.05. For the control group, no areas of significant differences in coherence between tasks were found for any other electrode pairs.

Within-group task comparisons at *P*_uncorrected_ <0.05 show that there were significant differences in interhemispheric coherence between faces and chairs tasks at various electrode pairs for the control group (increased coherence for chairs relative to faces at electrode pair TP7-TP8, decreased coherence for chairs relative to faces at electrode pairs FT7-FT8, TP7-TP8, P7-P8). However, such differences were only seen for one electrode pair in the ASC group (decreased coherence for chairs relative to faces at electrode pair T7-T8) (Figures 4 and 5). No within-group differences in coherence between tasks survived correction for multiple comparisons at *P*_FDR-corrected_ <0.05.

### Power analysis

No significant effects of group (F_1, 28_ = 1.911, *P* = 0.178) or task (F_1, 28_ = 2.240, *P* = 0.146) were found. Group-by-frequency band (F_1.003; 28.088_= 2.392, *P* = 0.133) and task-by-frequency band (F_1.001; 28.017_ = 2.329, *P* = 0.138) interactions were also not significant.

## Discussion

Coherence is an important tool for the study of complex cortical network dynamics and temporal fluctuations in the coupling between neural signals. Previous studies have shown that measures of coherence reflect patterns of cortical connectivity in the brain and that decreased values of coherence are associated with reduced connectivity between distant neural networks [41,57].

The results of the present study show a widespread and consistent reduction in interhemispheric coherence in the ASC group compared to the control group, during both visual tasks. These group differences are spread across the entire time-frequency spectrum, though they are more pronounced at frequencies lower than about 13 Hz and generally around 150 ms post-stimulus onset (Figures 1 and 2 – uncorrected for multiple comparisons). We hypothesize that these results are indicative of an overall impairment in functional interhemispheric connectivity during visual processing in people with ASC. This hypothesis is supported by previous reports of decreased structural and functional interhemispheric connectivity in ASC [14,15,22-24]. In addition, the tasks employed in this study involved object categorization, with participants needing to decide whether each presented image was of a chair or a human face. There is evidence that object categorization may be impaired in people with ASC [58-60]. It is also interesting to note that superordinate distinctions in object categorization can occur relatively soon after stimulus presentation. Van Rullen and Thorpe [61] reported electrophysiological differences associated with superordinate categorical differences (for example, animals vs. vehicles), peaking between 200 and 250 ms post-stimulus onset in typical controls. Similarly, Curran *et al*. [62] have reported ERP data indicating that feature analysis (supporting the process of finding similarities that link object exemplars into categories) precedes later processing stages associated with recognition of specific objects. Hence, it can be hypothesized that the relatively reduced coherence manifested in the current study by the participants with ASC from around 150 ms is related to atypical performance in categorization.

Consistent with this hypothesis, studies of the time-frequency responses of typically developing members of the general population to visual stimuli, including houses and faces, have shown that these responses could be explained by amplitude increases maximal in the 5 to 15 Hz frequency band, between 100 and 200 ms post-stimulus onset [63,64]. These reported frequencies are similar to the ones at which the participants with ASC display decreased interhemispheric coherence in the current study. The paradigms employed by Rousselet *et al*. [63] and Tang *et al*. [64] differ significantly from the one used in the current study, and neither explored coherence of EEG activity between different electrode sites. Nevertheless, it is interesting to consider their results in light of the present study, where most group differences in interhemispheric coherence are found in a frequency band below around 13 Hz – the differences in coherence observed in the current study may relate to differences in brain activity associated with structural encoding of the observed images as part of their initial categorization as either faces or chairs. While no correlations were found between AQ or IQ and coherence for either the ASC or the control group, it is important to note that the power to detect a correlation is low given the small sample size in the current study. It is interesting to note, however, that in the ASC group the correlations between coherence and AQ scores were negative, for both tasks and all electrode pairs.

Observation of Figures 1 and 2 also seems to indicate that during the chair task, but not the face task, there is relatively decreased interhemispheric coherence in the ASC group earlier in the response window (less than 200 ms post-stimulus onset), at higher frequencies (>13 Hz), in more posterior regions of the cortex. While this observation is interesting, these group differences do not survive correction for multiple comparisons and without additional group-by-task interactions analyses further discussions on the interpretation of these findings would be speculative. Informed by the methods of previous WTC analysis of EEG data [52] and taking into account the small sample size of the current data set, in the current study it was decided to run the statistical analysis in a non-parametric context. In this case, group-by-task interaction analyses are not trivial to perform, and algorithms for non-parametric interaction analyses are still under development [65,66].

In the current study, the lack of significant differences in EEG power spectra between groups or tasks also establishes a distinction between coherence measures and power spectrum analysis; changes in coherence values are not a reflection of changes in EEG power spectra in any frequency band. This is in accordance with some previous studies reporting the absence of abnormal patterns in EEG power spectra in individuals with ASC [67,68].

While there are clear differences in interhemispheric coherence between the ASC and the control groups in this study, the small sample sizes limit the statistical power of the comparisons. Additionally, it is important to note that due to the size of the data matrices being analyzed in this study (36 frequency points by 401 time points giving a total of 14,436 data points) standard methods for correction for multiple comparisons, such as Bonferroni, were not suitable. However, despite the fact that previous studies using WTC for the analysis of EEG data do not correct for multiple comparisons [52], care must be taken when interpreting uncorrected statistical results. Although significant group differences are seen in well-defined time-frequency clusters, increasing the likelihood of these differences being meaningful [69], correction for multiple comparisons was still performed.

The multiple comparison problem for such a large data set (a large volume of data per participant, despite a low sample size) must be carefully considered [70]. As mentioned above, conservative methods, such as Bonferroni correction, are less suitable as they lead to a high number of Type II (false-negative) errors, potentially losing true differences. However, the absence of any type of correction leads to the presence of Type I (false-positive) errors. Less conservative methods, such as False Discovery Rate correction (FDR; [55]), are commonly used in the statistical analysis of functional neuroimaging data, usually comprising hundreds of thousands of data points, and so FDR was considered suitable for use in the current study. However, it is important to note that as highlighted in a review on Type I and Type II error concerns in neuroimaging research [71], even FDR correction may be overly conservative when dealing with small effects. In a review by Lieberman and Cunningham [71], it is suggested that systematic meta-analyses should be used as an alternative approach in dealing with type I and type II errors, given that these random errors should not replicate across multiple studies, unlike true significant effects.

In the present study the only group difference that survives FDR correction is an area of decreased coherence for the ASC relative to the control group, for electrode pair T7-T8 in the faces task. This area is located at around 300 ms post-stimulus onset, on a frequency band between 7 and 10 Hz. It is interesting to note that temporal sites have previously been associated with visual processing of faces, albeit at earlier post-stimulus onset times [64,72]. The measures used in these investigations differ from the one used in the current study: the former uses measures of localized brain activity indexed by ERP components, while the latter uses a measure of interhemispheric coherence. Nevertheless, these previous investigations provide evidence that temporal regions are functionally involved in facial processing and it can be hypothesized that the group differences identified in the present study reflect atypical face processing in people with ASC, indexed by decreased interhemispheric coherence between temporal sites in this group.

As pointed out by Srinivasan *et al*. [73], moderate to large EEG coherence can also arise from volume conduction effects. However, in the current study, the finding of specific time-frequency regions surviving FDR correction suggests that group differences in interhemispheric coherence are not simply the result of differences in magnitude of volume conduction between the two groups, but represent a difference in genuine source coherence. Similarly, previous studies have shown that reference electrodes may influence coherence calculations of EEG signals [74]. Of particular interest to the current study are the findings of Essl *et al*. [74], showing that reference signals originating from a nose reference electrode may artificially increase coherence values. However, in the current study the reference electrode was the same for all individuals, and it is reasonable to assume that group and task differences in coherence would not be affected by the choice of reference electrode. It is also important to note that although group differences surviving FDR correction are quite limited, considering the small population size of the current study and taking into account the review by Lieberman and Cunningham [71] mentioned above, it is possible that in this case the FDR correction may have been overly conservative, and that other equally important, but small, effects are being missed.

Decreased interhemispheric coherence in ASC has been reported in previous studies [40,41]. Isler and colleagues [41] found decreased interhemispheric synchrony in children with ASC, when compared to typically developing children, in occipital lobes, in and below the theta frequency band (<8 Hz), during a visual stimulation task. In an investigation of resting state EEG coherence in children with ASC, Coben *et al*. [40] found evidence of decreased interhemispheric delta (0 to 4 Hz) and theta (4 to 8 Hz) coherences in frontal regions, as well as decreased delta, theta and alpha (<13 Hz) interhemispheric coherences in temporal areas of the cortex. Coben *et al*. [40] also report a decrease in delta, theta and beta (<8 Hz and 13 to 30 Hz) interhemispheric coherences in parietal regions of the brain. Although the paradigms and population samples of the current study and those of Isler *et al*. [41] and Coben *et al*. [40] are not directly comparable (in that the current study was an investigation of task related coherence in adults and the others examined resting state and visual flash evoked coherence in children), all studies investigated a variety of brain regions and frequency bands, and the results of the current study can be considered as supported by and complementary to those of Isler *et al*. [41] and Coben *et al*. [40]. Additionally, the investigation of functional brain coherence using other modalities confirms that decreased interhemispheric connectivity in people with ASC is a consistent finding [75,76]. In a resting state MRI study that recruited individuals with and without ASC from late childhood to early adulthood, Anderson *et al*. [76] found evidence of impaired interhemispheric connectivity in ASC in sensorimotor cortex, anterior insula, fusiform gyrus, superior temporal gyrus and superior parietal lobule, while Dinstein *et al*. [75] investigated interhemispheric coherence in toddlers with ASC using MRI, and reported decreased interhemispheric connectivity in putative language areas, such as superior temporal gyrus.

The present study investigated task-related interhemispheric coherence during visual perception of chairs or faces in cortical regions, including frontal, temporal and parietal areas. These disparate regions are likely to have been involved in a variety of different components of the task, from visual processing to visual categorization learning [77-79]. The relatively extensive analysis of coherence performed in the current study, over a time-frequency range from 5 to 40 Hz and 1 to 400 ms post-stimulus onset, supports the conclusion that interhemispheric connectivity in ASC is impaired not only in posterior regions but also in frontal and temporal regions of the cortex (as reflected by group differences not corrected for multiple comparisons), in similarity to the results of the resting state studies of Coben *et al*. [40], Dinstein *et al*. [75] and Anderson *et al*. [76]. In addition, the use of the WTC approach enabled evidence to be gathered suggesting that it was in lower frequency bands that group differences in EEG responses to the tasks were concentrated, as shown by the uncorrected group differences’ results. Previous studies have shown evidence relating low frequency theta and alpha synchronization with top-down working memory processes, subserving functional integration over multiple neural networks [80,81]. The visual matching task included in the current study can be considered to involve working memory processes [81,82], and we hypothesize that the decrease in low frequency coherence in the ASC group reflects atypical neural connectivity that results in an impairment of integration of information across neural networks. Additionally, previous studies have suggested the existence of a relation between the size and distance of a neural interaction and the frequency of the neural synchronization. In particular, it has been reported that lower frequency oscillations seem to be associated with larger neuronal assemblies and long range connectivity [80,83-86]. The results of the present study are complementary to these reports, and show further evidence supporting theories of impaired long range connectivity in ASC [9,10,14,15]. Our results suggest that interhemispheric connectivity in ASC is widely atypical, and it is hypothesized that this may have greater implications for tasks that require integration of information over neural networks spread across both cortical hemispheres.

As can be seen in Figures 4 and 5, some differences were found in within-group interhemispheric coherence between the chairs and the faces task, for both groups (uncorrected for multiple comparisons). Differences in coherence between tasks were not constrained to a particular region of the time-frequency map, occurring at both early (50 ms) and late (300 ms) post-stimulus onset times, from lower (7 Hz) to higher (23 Hz) frequencies. These differences were significant at a larger number of electrode pairs for the control group than for the ASC group, possibly reflecting an impairment in task differentiation in people with ASC relative to typically developing controls. This is consistent with previous investigations showing impairments in object categorization and face processing in people with ASC [43,44,59,60]. It is also consistent with the results from an ERP investigation using the same paradigm as the current study [45]. It is important to note that although within-group differences in coherence between tasks were found, these were not as significant as the group differences represented in Figures 1 and 2, and did not survive correction for multiple comparisons. This may be related to the behavioral results of this study, showing an absence of significant group differences in task performance in terms of speed and accuracy of image recognition. However, behavioral results also show that across both groups, the face task was performed a little less accurately than the chair task. This task effect in accuracy was driven by relatively lower accuracy for the ASC group in the face task, reflected in a group-by-task interaction that approached significance (F_1, 28_ = 3.661, *P* = 0.066). Despite this, both the ASC and the control groups performed the tasks with high degrees of accuracy and close to ceiling level (Table 2). The trend observed in the group-by-task interaction is probably the result of the majority of control subjects performing at ceiling level, and the participants in the ASC group making a larger, yet still small, number of mistakes. Clinically, these differences are not considered to be relevant, as the ASC group still presents accuracy scores of around 90% for the face task. The paradigm used in the current study may thus not have been sufficiently demanding to detect possible group differences in task performance or task differences in coherence. Further research is recommended to examine potential correlations between specific cognitive or behavioral functions and atypical patterns of interhemispheric coherence in people with ASC. Additionally, future investigations using the WTC algorithm should seek to improve statistical power of their analyses by using larger population sizes and correcting for multiple comparisons using FDR or similar method.

## Conclusions

The results of the current study support the potential value of WTC in examining the time-frequency microstructure of task-related interhemispheric EEG coherence in people with ASC. Using WTC, we showed that interhemispheric coherence is reduced in people with ASC, in a time and frequency specific manner, during visual perception and categorization of both social and inanimate stimuli, and that this reduction in coherence is widely dispersed across the brain.

## Availability of supporting data

The data sets supporting the results of this article are included within the article and its additional files (Additional file 1, Additional file 2, Additional file 3 and Additional file 4).

## Abbreviations

ANOVA: Analysis of Variance; AQ: Autism Quotient; ASC: Autism Spectrum Conditions; EEG: Electroencephalography; ERP: Event-Related Potential; FDR: False Discovery Rate; FFT: Fast Fourier Transform; IQ: Intelligence Quotient; MRI: Magnetic Resonance Imaging; WASI: Wechsler Abbreviated Scale of Intelligence; WTC: Wavelet Transform Coherence.

## Competing interests

The authors declare that they have no competing interests.

## Authors’ contributions

AC carried out the data analysis, contributed to the interpretation of results and drafted the manuscript. AA contributed to the data analysis and interpretation of results. OC contributed to study design and data acquisition, as well as to the interpretation of results. APW contributed to the statistical analyses. SBC participated in study design and coordination. HR participated in study design and coordination, contributed to interpretation of results and helped draft the manuscript. All authors read and approved the final manuscript.

## Supplementary Material

Additional file 1 Raw EEG data for the chair task (part 1). Click here for a file

Additional file 2 Raw EEG data for the chair task (part 2). Click here for a file

Additional file 3 Raw EEG data for the face task (part 1). Click here for a file

Additional file 4 Raw EEG data for the face task (part 2). Click here for a file
